# A Metabolomics-Based Strategy for the Quality Control of Traditional Chinese Medicine: Shengmai Injection as a Case Study

**DOI:** 10.1155/2013/836179

**Published:** 2013-01-20

**Authors:** Xiaodong Li, Huiyuan Chen, Wei Jia, Guoxiang Xie

**Affiliations:** ^1^College of Pharmacy, Fujian University of Traditional Chinese Medicine, Fuzhou, Fujian 350108, China; ^2^Center for Translational Biomedical Research, University of North Carolina at Greensboro, North Carolina Research Campus, Kannapolis, NC 28081, USA

## Abstract

Quality control of traditional Chinese medicines (TCMs) used clinically is becoming a challenge and has limited the development of TCM due to the high variability in concentration levels of active ingredients and markers as well as the lack of well-established criteria. Using Shengmai injection, which is a well-established TCM, as an example, we developed an integrated profiling approach that simultaneously captures the entire spectrum of ingredients and quantitatively determines the levels of seven key ingredients in the TCM product. A multivariate statistical model was constructed to establish a “seven-marker-” based quality standard that qualified the majority of samples in this study. This newly developed strategy showed that a panel of key ingredients or markers in the TCM product were relatively consistent within a statistically acceptable range. Therefore, this metabolomics-based approach will complement the current quality control standard using the concentration of several key ingredients or their total content and help improve the consistency and clinic efficacy of TCM products.

## 1. Introduction

Traditional Chinese medicine (TCM) is gaining greater acceptance throughout the world, especially in western countries, for improving health and preventing or healing diseases. Recently, a traditional herbal medicinal product manufactured in China called Diao Xin Xue Kang, used for treating myocardial ischemia, has been approved for sale in Europe [1]. TCMs are composed of more than one herb, and the quality and content of the herbs are highly variable depending on geographical origins, climate, cultivation, and the growth stage when harvested [2]. The therapeutic effect of TCM is based on the synergic effect of its complex components, which is different from that of western medicine [3–6]. However, there is currently insufficient data on TCM due to the lack of modern and scientific approaches for standardization. Traditional methods for determining the quality and authenticity of a complex herbal medicine or preparation are to assess a defined number of bioactive components or pharmacological constituents [7]. Quality-control of TCMs continues to be a challenge and restricts the development of TCM throughout the world. Current criteria listed in pharmacopeia or other criteria issued by the Ministry of Health of China usually use the concentration of several key ingredients or their total content to control the quality and ensure reproducible clinical efficacy. Although the concentration of each bioactive component or the total concentration of all components may meet the requirements of the criteria, there is often variation in the concentration of each of these bioactive components between batches. Furthermore, in some cases one or more of the bioactive components may be absent from a preparation, but the total content can still meet the criteria stipulated. It is worth noting that different concentrations of the proportion of mass constituents in herbal medicine may result in different therapeutic effects [8]. For example, EGb761, a *Ginkgo* biloba extract produced by Schwabe Pharmaceuticals, was standardized on the basis of the presence of 24% (w/w) “*Ginkgo *flavone” glycosides and 6% (w/w) terpene lactones. However, *Ginkgo *extracts from other manufacturers easily met this standard, but were clearly distinct from EGb761 [9]. Xie [6] reported that it was difficult to distinguish between the EGb761 standard and other preparations of *Ginkgo *extracts containing rutin. Therefore, it is challenging to obtain well-quality-controlled herbal drugs and preparations with current criteria/standards.

Over the past several decades, the chromatographic fingerprint technique has been used as a more accurate approach for controlling the quality of herbal medicines or their products due to the systemic characterization of compositions of samples and focusing on identifying and assessing the stability of the plants. The fingerprint analysis technique has been introduced and accepted by the World Health Organization (WHO) [10], Food and Drug Administration (FDA) [4], and European Medicines Agency (EMEA) [5] as a strategy for assessing consistency between batches of botanical drugs. In 2004, the State Food and Drug Administration (SFDA) of China also required that all injections made from herbal medicines or their raw materials should be standardized by chromatographic fingerprinting [11]. However, a validated fingerprint method has not been accepted in general quality control standards of herbal medicines until now. One of the main difficulties is the lack of an analytical method for scientifically evaluating the complex chromatograms of herbal medicines [9]. The technique uses the traditional concept of pharmaceutical analysis and combines it with an analysis of chromatographic peaks. However, the current fingerprint analysis technique does not correct for chromatographic shifts among different runs or from different experimental conditions and cannot compare the fingerprints of TCMs from different species of herbs, grown at different locations, from different harvesting seasons, or extracted and processed using different methods. Therefore, an effective quality control method for TCMs or herbal medicines should be developed in order to further develop their standardization and modernization. 

Metabolomics, or metabonomics, which aims to identify and quantify the full complement of low molecular weight (<1,000 Da), soluble metabolites in actively metabolizing tissues [12, 13], has garnered extensive awareness and interest in the research community, especially in botanical studies for the quality control of plants [14–17], metabolic phenotyping [18, 19], and the pharmacological effects of TCMs [20, 21]. A metabolomics approach is uniquely suited for developing a new and effective method for the quality control of the processing and manufacturing of TCMs. The central goal of our strategy is to ensure that representative bioactive components in one preparation are consistent, that the content of each bioactive component meets the stipulated standards, and that concentration proportions of mass constituents are within a reasonable range. Here, we reported a quality control method by integrating the metabolic profiling approach together with the multivariate statistical analysis on the study of a well-quality-controlled injection, Shengmai [22], prepared from *Radix Ginseng*, *Radix Ophiopogonis,* and *Schisandra chinensis*. A total of 22 Shengmai injections obtained from 4 different manufacturers in China were analyzed by liquid chromatography/time-of-flight mass spectrometry (LC-TOF-MS) to obtain a metabolic profile. Saponins from the principal herb (Jun Yao in Chinese) under the theory of TCM,* Radix Ginseng*, were quantitatively measured to determine whether a given batch met established acceptance criteria or our newly established standard as well as the content variation among different batches. Eight ginsenosides, namely, Rb_2_, Rd, Rf, Rg_2_, Rg_1_, Rb_1_, Rc, and Re, were analyzed according to the method described in our previous study [17].

## 2. Materials and Methods

### 2.1. Chemicals and Materials

HPLC grade acetonitrile, methanol, and formic acid were obtained from Sigma Aldrich (St. Louis, MO, USA). Ultrapure water was prepared using a Milli-Q system (18.2 MΩ, Milford, MA, USA). Shengmai injection samples were obtained from four different manufacturers from China (sample A, B, C, and D with several batches of each; Table 2). Ginsenoside Re, Rb1, Rc, Rb2, Rd, Rg2, Rg1, and Rf standards were obtained from ChromaDex (Irvine, CA, USA). 

 Individual stock solutions (1.0 mg/mL) of eight standard ginsenosides were prepared in methanol for LC-TOF-MS analysis. 

Working standards (5, 10, 20, 50, 100, 200, 500, 1000, 2000, 5000, and 10000 ng/mL) were prepared by diluting the corresponding stock solution with methanol for the LC-TOF-MS analysis. All stock solutions were stored under refrigerated conditions (4°C).

### 2.2. Sample Preparation

A 10 *μ*L sample of each batch of Shengmai injection was diluted to 1 mL in ultrapure water. The solutions were then vortexed for 30 s, filtered through a syringe filter membrane (0.20 *μ*m), and then injected directly into the LC-TOF-MS system.

### 2.3. HPLC-TOFMS Analysis

An Agilent HPLC 1200 system equipped with a binary solvent delivery manager and a sample manager (Agilent Corporation, Santa Clara, CA, USA) was used with chromatographic separations performed on a 4.6 × 150 mm 5 **μ**m Agilent ZORBAX Eclipse XDB-C18 chromatography column. The LC elution conditions were optimized as follows: isocratic at 1% B (0–0.5 min), linear gradient from 1% to 20% B (0.5–9.0 min), 20%–75% B (9.0–15.0 min), 75%–100% B (15.0–18.0 min), isocratic at 100% B (18–19.5 min); linear gradient from 100% to 1% B (19.5–20.0 min) and isocratic at 1% B (20.0–25.0 min). Here, A = water with 0.1% formic acid and B = acetonitrile with 0.1% formic acid. The column was maintained at 30°C. A 5 **μ**L aliquot sample was then injected onto the column. Mass spectrometry was performed using an Agilent model 6220 MSD TOF mass spectrometer equipped with a dual sprayer electrospray ionization source (Agilent Corporation, Santa Clara, CA, USA). The system was tuned for optimum sensitivity and resolution using an Agilent ESI-L low concentration tuning mix in both positive (ES+) and negative (ES−) electrospray ionization modes. The Agilent API-TOF reference mass solution kit was used to obtain accurate mass TOF data in both positive and negative mode operations. The TOF mass spectrometer was operated with the following optimized conditions: (1) ES+ mode, capillary voltage 3500 V, nebulizer 45 psig, drying gas temperature 325°C, drying gas flow 11 L/min, and (2) ES− mode, similar conditions as the ES+ mode except that the capillary voltage was adjusted to 3000 V. The TOF mass spectrometer was calibrated routinely in ES+ and ES− modes using the Agilent ESI-L low concentration tuning mix. During metabolite profiling experiments, both plot and centroid data were acquired for each sample from 50 to 1,000 Da over a 25 min analysis time.

### 2.4. Data Analysis

The resulting.d files were centroided, deisotoped, and converted to mzData xml files using the MassHunter Qualitative Analysis Program (vB.03.01) (Agilent). Following conversion, xml files were analyzed using the open source XCMS package (v1.24.1) (http://metlin.scripps.edu/), which runs in the statistical package R (v.2.12.1) (http://www.r-project.org/), to pick, align, and quantify features (chromatographic events corresponding to specific *m/z* values and retention times). The software was used with default settings as described (http://metlin.scripps.edu/) except for xset (bw = 5) and rector (plottype = “m”, family = “s”). The created.tsv file was opened using Excel software and saved as an  .xls file. The resulting 3D matrix containing arbitrarily assigned peak index, retention time, and abundance value (.xls file) were then exported to SIMCA-P software 12.0 (Umetrics, Umeå, Sweden) for multivariate statistical analysis. Mean-centered and par-scaled (scaled to square root of SD) mathematical methods were performed to pretreat the datasets that were obtained from the previous samples. Principal component analysis (PCA) was used to visualize general clustering, trends, and outliers among the observations. 

### 2.5. Strategy for Quality Control

**Figure 1:**
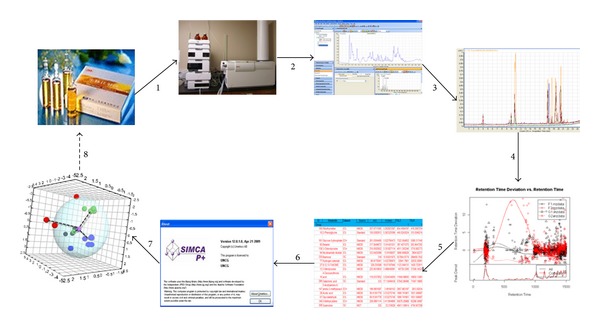
Schematic diagram of the strategy used for the quality control of traditional Chinese medicine (TCM). (1) LC-TOF-MS analysis of TCM; (2) Pretreatment data obtained from the LC-TOF-MS analysis; (3) Data export using software; (4) Exported.xml data files are analyzed using the open source XCMS package to pick, align, and quantify features; (5) The resulting 3D matrix contains arbitrarily assigned peak index, retention time, and abundance value (.xls file); (6) The  .xls data files are then exported to SIMCA-P software 12.0 (Umetrics, Umeå, Sweden) for multivariate statistical analysis; (7) A quality control standard is then established; (8) The established standard is used for quality control of TCM.

An integrated metabolomics and multivariate statistical analysis was conducted according to the process as proposed in Figure 1. Representative Shengmai injections were used as a model TCM for analysis and characterization. LC-TOF-MS was used to measure the global metabolite pool of the samples and quantify the bioactive ginsenosides.

 The metabolome of the herbal medicine was obtained from the metabolomics analysis. The metabolite similarities were visualized from the PCA analysis. Quantitative analysis of the reported key ingredients was performed, and the concentration of each key ingredient had to meet the requirements of the criteria. Furthermore, the concentration proportion of each key ingredient had to be within a reasonable range to maintain consistency in pharmacological effects and clinical efficacy between batches. The spatial distance away from the standard center also had to be within a reasonable range to ensure the quality, and the range was set logically based on the data. For example, if the concentration was within a range of 85%–115% of the standard concentration, then the sample dot would be within the sphere and identified as qualified product. However, if the concentration was out of range from the standard concentration, then the red dots would be outside of the sphere and the preparation would be considered as an unqualified product. The standard locus of a TCM can be dotted using SIMCA-P software by importing data obtained from a sample made from the standard herbs using the preparative procedure. As evident in Figure 1, the violet dot represents the standard criteria, and the group/products expressed as green, black, and blue dots are within the scope of acceptable quality. In contrast, the red dots represent samples not meeting the quality criteria. 

## 3. Results and Discussion

### 3.1. Method Validation

**Figure 2:**
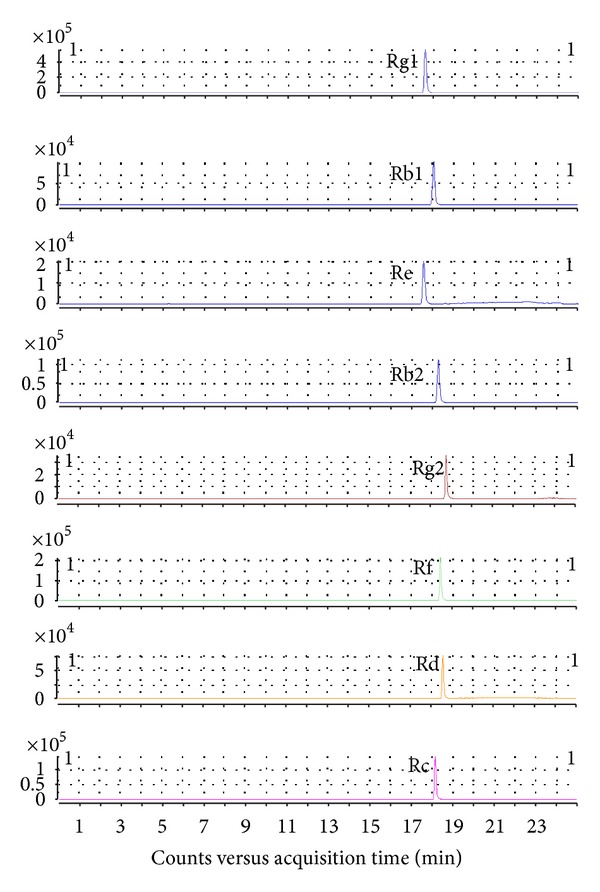
Extracted ion chromatograms of eight ginsenosides, including Rg1, Rb1, Re, Rb2, Rg2, Rf, Rd, and Rc.

Extracted ion chromatograms of eight ginsenosides, including Re, Rb1, Rc, Rb2, Rd, Rg2, Rg1, and Rf, are shown in Figure 2. Calibration curves of peak area (*y*) versus concentration (*x*) were constructed. These curves showed good linearity over the concentration range listed in Table 1 with correlation coefficients from 0.9971 to 0.9997, respectively. The limit of detection (LOD) and limit of quantitation (LOQ) were determined using the signal-to-noise ratio (*S/N*) provided by MassHunter quantitative analysis software (Ver. B.01.04), and all of the *S/N* values were greater than 10.0, which allowed for the individual calculation of each compound's quantification limit. The reproducibility was investigated using the spiked standard solution. After correction with the internal standard, the relative standard deviations (RSDs) of the peak area for each spiked standard were comparable, with RSDs below 10%. In addition, the stability assay was evaluated using the aforementioned spiked standards. For most spiked compounds, the RSDs were less than 6.5% within 24 h and less than 10% within 36 h. To further validate our methodology for analyzing complex endogenous metabolites, each 20 *μ*L aliquot of 0.5, 1, and 2 *μ*g/mL of the spiked standard solution was mixed with a 20 *μ*L aliquot of diluted Shengmai injection. Recovery was calculated using the aforementioned calibration curves, and the mean recoveries of all these compounds ranged from 90% to 110%, with RSDs better than 15%. 

### 3.2. LC-TOF-MS Analysis of Shengmai Samples

**Figure 3:**
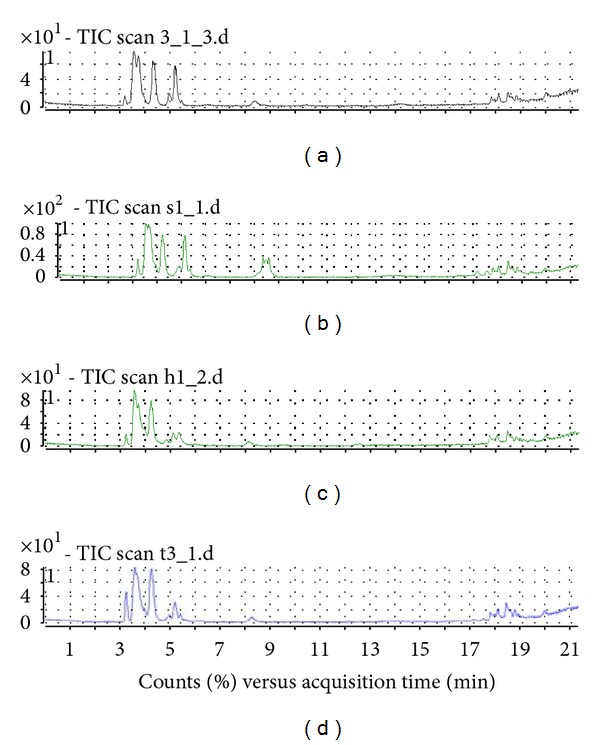
Total ion current (TIC) chromatograms of four Shengmai injection samples.

LC-TOF-MS analysis was then performed for 22 Shengmai samples obtained from four manufacturers. Typical LC-TOF-MS chromatograms of the samples are shown in Figure 3, where similar characteristic peaks were observed, with the exception of some minor peaks. This finding may indicate that the components of each injection manufactured by different factories are similar, except for the quantity of the components contained within each. In particular, the peaks located between 17 and 19 min, which corresponded to the saponins in the principal herb, *Radix Ginseng*, showed very similar profiles. 

### 3.3. Quantitative Analysis of Ginsenosides in Shengmai Samples

Using our optimized LC-TOF-MS method, a high regression coefficient (*r* > 0.99) value for each calibration curve from the eight spiked standards was obtained, indicating good linearity in this study (Table 1). The quantitative analysis results of ginsenosides in different batches of one manufacturer and from different manufacturers are listed in Table 2. The content of Rg2 was relatively low in the injections and below the LOQ, and therefore only seven ginsenosides were quantified (Table 2). The levels of the seven ginsenosides from all samples were comparable and met the criteria requirements issued by the Ministry of Health of China, which stipulates that the level of Rg1 should be higher than 80 ng/mL and the Re level should be at least 40 ng/mL [22]. However, the concentrations of Rg1 and Re were significantly different between several batches form one manufacturer as well as between different manufacturers, though they all meet the criteria for clinical use. However, due to the differences in ginsenoside concentrations in preparations from the four manufacturers, differences in clinical efficacy would be expected. Furthermore, the variations in concentration between batches were large, indicating that the reproducibility of the product was poor. Because the preparative procedure was standardized, the differences may be a result of variability in the raw materials used. Therefore, it is very important to control the quality consistency between factories, and particularly among the different batches, to ensure satisfactory quality and comparable efficacy between batches. 

### 3.4. Principal Component Analysis

**Figure 4:**
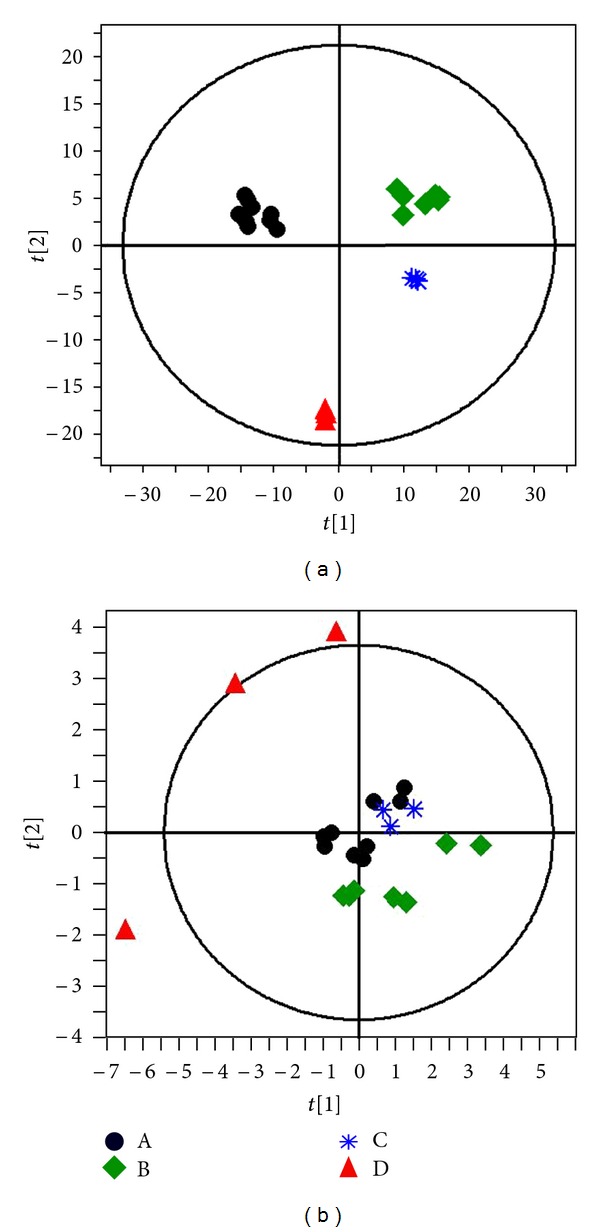
PCA sores plot of (a) metabolic profile of 22 Shengmai injection samples using all of the data and (b) metabolic profile of 22 Shengmai injection samples using the ginsenoside quantitative data.

Chemometrics, the science of extracting information from chemical measurement systems with mathematics and statistics, can be employed to the multicomponent metabolic profile patterns to ascertain consistency/variations in multiple batches of herbal medicines. Application of chemometrics methods to multiple commercial batch metabolic profile data is a big leap forward in the quality assurance of medicinal herb preparations which provides information on batch consistency, “outlier” batches, and possible “contaminants.” One impressive example of exploratory data analysis is the PCA which is a “workhorse” in chemometrics [23]. PCA, unsupervised clustering method requiring little prior knowledge of the dataset, acts to reduce the dimensionality of multivariate data without losing important information [24], and supervised orthogonal partial least-squares-discriminant analysis (OPLS-DA) of the metabolomics data has been extensively used for the holistic quality inconsistencies of herbal medicines [21, 25]. In this study, both UV-scaled (scaled to the standard deviation) and par-scaled (scaled to the square root of the standard deviation) mathematical methods were performed to pretreat the datasets obtained from 22 batches of injections. In order to explain the difference and the consistency of Shengmai samples from 4 different manufacturers, the data obtained from all metabolites and the quantitative concentration of Shengmai samples shown in Table 2 were imported to SIMCA P 12.0 software to obtain the PCA scores plot. Clear separation of the four different samples was observed in the PCA scores plot of all data (Figure 4(a)) and quantitative ginsenoside data (Figure 4(b)), where each coordinate represents one sample. Using the quantitative analysis data, the red dots were derived from other samples and represent outliers; thus, they are considered to be unqualified products. 

### 3.5. Quality Control of Shengmai Injection

**Figure 5:**
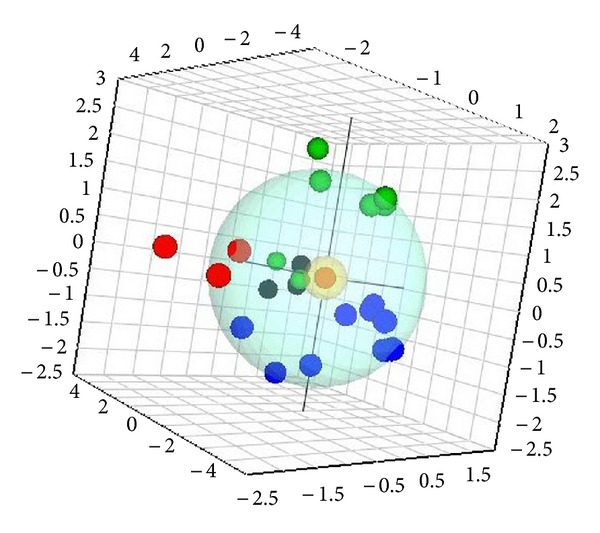
PCA scores plot of the ginsenoside metabolite profile of 22 Shengmai injection samples obtained from four different manufacturers. Blue dot: sample A; green dot: sample B; black dot: sample C; red dot: sample D.

Using the established approach, the quantitative analysis results of 22 batches of Shengmai samples (Table 2) were imported into SIMCA P 12.0 software to determine the quality control results. Using the standard compound concentration (the average concentration of seven ginsenosides of 22 batches of Shengmai samples) as the center, all of the qualified products would fall within the range of the spatial sphere (Figure 5) and the unqualified products would be outside the sphere. Sample C (black dots) met all of the requirements, sample A (blue dots) met most of the requirements, and samples B and D (green and red dots, resp.) were outside of the established standard. This clearly indicates that there were differences among products obtained from different manufacturers as well as different batches of product generated by one manufacturer. Using the established standard, some samples were found to be unqualified products, though they all met the current standard. Therefore, these data indicate that this method will help to guarantee the quality, reproducibility, and efficacy of the products made from natural herbs.

## 4. Conclusion

For complicated herbal products, it is easy to control the concentration of several key ingredients to achieve a standard level; however, it is difficult to keep the concentration proportion of different key ingredients in a comparable range. Different concentration proportions of mass constituents may result in different therapeutic effects of herbal medicines and subsequent variability in clinical efficacy. Our strategy proposed in this study will enable us to measure the global metabolite profile of preparations and simultaneously quantify the key ingredients in order to ascertain treatment efficacy and minimize probable side effects, which will greatly enhance pharmacological evaluation. We believe that this approach will help to prefect the current quality control method used for complicated herbal materials or medicines, improve the quality of TCMs, and ensure the efficacy of the products used in the clinic. 

## Figures and Tables

**Table 1:** Calibration curves of eight ginsenosides^a^.

Compound	Rt (min)	Linearity			LOD (ng/mL)	LOQ (ng/mL)	*S/N*
		Range (ng/mL)	Calibration curves	*R* ^2^			
Rg2	18.72	185.55–5834.39	*A* = 35.65*C* + 4737.90	0.9971	46.39	92.78	>25
Rf	18.41	92.78–5863.20	*A* = 207.73*C* + 19139.65	0.9990	23.20	46.39	>19
Rd	18.57	88.18–5977.96	*A* = 68.68*C* − 2068.57	0.9997	23.20	56.18	>10
Rc	18.17	46.39–5937.50	*A* = 138.13*C* + 1677.32	0.9997	11.6	23.14	>11
Re	17.59	185.55–5720.31	*A* = 27.08 + 10884.83	0.9884	46.00	93.01	>12
Rb1	18.04	46.39–5916.00	*A* = 128.06*C* + 3291.30	0.9994	11.58	23.20	>19
Rb2	18.24	92.78–5874.07	*A* = 149.19*C* + 7256.20	0.9989	23.20	46.39	>18
Rg1	17.59	185.54–5937.3	*A* = 707.444*C* + 113373.52	0.9929	46.38	92.77	>30

^a^Calibration curves were constructed based on the peak area (*A*) versus concentration (*C*). *R* ^2^: linearity correction coefficient within the linear range of the sample concentration.

**Table 2:** Quantitative analysis results of 7 ginsenosides in Shengmai samples obtained from different manufacturers.

Manufacturer	Batch	Rf (*μ*g/mL)		Rd (*μ*g/mL)		Rc (*μ*g/mL)		Re (*μ*g/mL)		Rb1 (*μ*g/mL)		Rb2 (*μ*g/mL)		Rg1 (*μ*g/mL)	
		Content	Mean	Content	Mean	Content	Mean	Content	Mean	Content	Mean	Content	Mean	Content	Mean
A	1	31.44	31.21	47.17	49.60	159.18	155.96	98.82	92.63	196.81	190.23	145.08	142.12	148.203	145.922
		31.95		47.67		155.86		90.08		186.46		142.04		146.519	
		30.24		53.95		152.83		89.00		187.43		139.24		143.045	
	2	36.14	36.40	59.00	60.15	170.96	174.31	82.60	86.51	228.21	233.92	155.92	159.17	165.648	169.609
		35.27		60.24		178.04		84.86		237.07		162.44		171.297	
		37.80		61.20		173.92		92.06		236.49		159.16		171.881	
	3	38.89	41.11	50.96	58.05	189.40	195.35	117.60	124.82	206.99	217.00	172.90	178.63	158.711	167.618
		42.08		68.24		192.80		124.60		217.83		176.81		167.248	
		42.36		54.94		203.84		132.27		226.18		186.18		176.894	

B	1	37.89	37.89	76.92	76.92	199.80	199.80	84.00	84.00	338.82	338.82	182.46	182.46	148.997	148.997
	2	34.27	34.27	63.96	63.96	209.33	209.33	118.07	118.07	448.86	448.86	192.31	192.31	122.846	122.846
	3	41.94	42.20	67.33	70.24	248.78	259.36	115.49	122.86	311.92	330.06	227.53	237.27	185.855	190.272
		42.45		73.14		269.95		130.23		348.21		247.01		194.69	
	4	27.48	29.03	60.70	61.29	170.33	175.27	89.37	87.84	305.55	306.64	155.35	159.90	120.291	124.315
		30.87		61.27		179.75		88.26		312.04		164.01		125.437	
		28.74		61.89		175.74		85.88		302.32		160.33		127.219	

C	1	43.17	44.27	70.33	69.79	241.48	244.37	115.49	124.36	357.08	355.17	220.81	223.62	197.394	204.205
		44.74		71.95		245.26		136.14		351.39		225.08		211.019	
		44.89		67.09		246.38		121.44		357.05		224.98		204.203	

D	1	43.09	43.83	66.41	71.18	188.39	197.72	121.00	121.13	253.69	264.56	172.48	180.72	129.588	133.091
		40.79		69.18		200.84		116.26		262.77		183.42		130.592	
		47.61		77.95		203.94		126.12		277.22		186.27		139.092	
